# Evaluation of pregnancy and delivery in 13 women who underwent resection of a sacrococcygeal teratoma during early childhood

**DOI:** 10.1186/s12884-014-0407-x

**Published:** 2014-12-12

**Authors:** Marijke EB Kremer, Margot M Koeneman, Joep PM Derikx, Audrey Coumans, Robertine van Baren, Hugo A Heij, Marc HWA Wijnen, René MH Wijnen, David C van der Zee, Ernest LW van Heurn

**Affiliations:** Department of Pediatric Surgery, Maastricht University Medical Center, Maastricht, The Netherlands; Department of Gynecology and Obstetrics, Maastricht University Medical Center, Maastricht, The Netherlands; Department of Pediatric Surgery, University Medical Center Groningen, Groningen, The Netherlands; Pediatric Surgical Center of Amsterdam, Emma Children’s Hospital University Medical Center and VU Medical Center, Amsterdam, The Netherlands; Department of Pediatric Surgery, University Medical Center Nijmegen, Nijmegen, The Netherlands; Department of Pediatric Surgery, Sophia Children’s Hospital, Erasmus University Medical Center Rotterdam, Rotterdam, The Netherlands; Department of Pediatric Surgery, Wilhelmina Children’s Hospital, University Medical Center Utrecht, Utrecht, The Netherlands

**Keywords:** Sacrococcygeal teratoma, Sequelae, Pregnancy, Vaginal delivery

## Abstract

**Background:**

Sacrococcygeal teratoma resection often brings changes in pelvic anatomy and physiology with possible consequences for defecation, micturition and sexual function. It is unknown, whether these changes have any gynecological and obstetric sequelae. Until now four pregnancies after sacrococcygeal teratoma resection have been described and cesarean section has been suggested to be the method of choice for delivery. We evaluated the pregnancy course and mode of delivery in women previously treated for a sacrococcygeal teratoma.

**Methods:**

The records of all patients who underwent sacrococcygeal teratoma resection after 1970 in one of the six pediatric surgical centers in the Netherlands were reviewed retrospectively. Women aged 18 years and older were eligible for participation. Patient characteristics, details about the performed operation and tumor histology were retrieved from the records. Consenting participants completed a questionnaire addressing fertility, pregnancy and delivery details.

**Results:**

Eighty-nine women were eligible for participation; 20 could not be traced. Informed consent was received from 41, of whom 38 returned the completed questionnaire (92.7%). Thirteen of these 38 women conceived, all but one spontaneously. In total 20 infants were born, 17 by vaginal delivery and 3 by cesarean section, in one necessitated by previous intra-abdominal surgery as a consequence of sacrococcygeal teratoma resection. Conversion to a cesarean section was never necessary. None of the 25 women without offspring reported involuntary childlessness.

**Conclusions:**

There are no indications that resection of a sacrococcygeal teratoma in female patients is associated with reduced fertility: spontaneous pregnancy is possible and vaginal delivery is safe for mother and child, irrespective of the sacrococcygeal teratoma classification or tumor histology.

## Background

**Figure 1:**
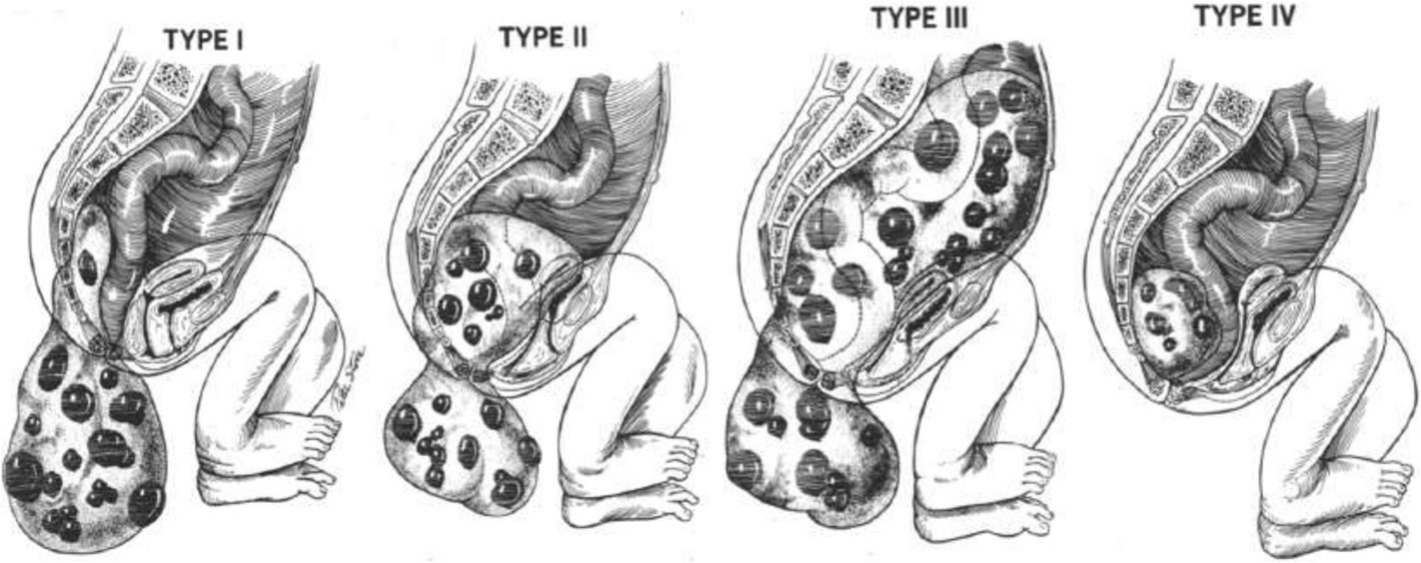
**Altman classification of sacrococcygeal teratomas** [6]**.**

Sacrococcygeal teratoma (SCT) is the most common tumor in infancy with a reported incidence between one in 15,000 to 40,000 live births and a female to male ratio of 4:1 [1-5]. Four types are distinguished according to the Altman classification, which describes the intra- and extra-pelvic extension of the tumor mass (Figure 1) [6]. Early resection with the coccyx en bloc is the standard treatment to prevent malignant transformation of these usually benign tumors [7-9]. Both the pressure of tumor mass and extended pelvic surgery in childhood may be associated with gynecological and obstetric sequelae.

SCT resection during childhood has been associated with various anatomical changes of the pelvis and pelvic cavity, such as a hypo-plastic sacral bone and spondylolysis of the fifth lumbar vertebra [10]. In addition, there is iatrogenic damage of the pelvic anatomy as in almost all cases the tumor is completely resected together with the coccyx and several perineal and pelvic muscles are fully or partly resected [8]. In women this may lead to pelvic functional changes such as organ prolapse [11].

It is unknown if the changes of the female pelvic anatomy and function, which may result in alteration of the birth channel, influence fertility, pregnancy and delivery. Until now, four cases of pregnancy after SCT resection have been described [12-14]. Cesarean section has been suggested to be the method of choice for delivery due to no progression of descend during childbirth [12,13]. Others reported an uncomplicated spontaneous vaginal delivery of a healthy term baby [14].

The aim of this retrospective multicenter study was to evaluate fertility, pregnancy and delivery in women treated for SCT during childhood.

## Methods

### Patients and methods

After ethical approval by the medical ethical committee of the Maastricht University Medical Center, the records of all patients treated for SCT after 1970 in one of the six Dutch pediatric surgical centers (Emma Children’s Hospital University Medical Center and VU Medical Center Amsterdam, University Medical Center Groningen, Maastricht University Medical Center, St. Radboud University Medical Center Nijmegen, Sophia Children’s Hospital Rotterdam and Wilhelmina Children’s Hospital Utrecht) were retrospectively reviewed and women aged 18 years and older at the time of data collection were eligible for participation.

Patient characteristics including age, sex, tumor histology, Altman-classification [6] and operation details were retrieved from the records. A self-designed questionnaire addressed the following issues: medical history, number of pregnancy attempts, completed pregnancies, involuntary childlessness, mode of delivery, obstetric intervention during delivery and details about possible perineal rupture. Furthermore the newborn’s characteristics gestational age, birth weight and neonatal condition post-delivery (good – moderate – bad) were assessed.

All patients were initially contacted with an information letter sent to the most recent address explaining the background of the study together with an informed consent form. Those who not responded were contacted two weeks later by telephone and by post. The questionnaire was sent after the signed informed consent had been returned.

### Statistical analysis

To test for baseline differences between women with intention to become pregnant and those with no intention to become pregnant, Fisher’s exact test was applied for categorical variables (Altman classification, tumor histology and coccyx resection). Mann–Whitney U test was applied to test for differences in age distribution between both groups. P-values of ≤ 0.05 were considered to be statistically significant. Statistical analysis was performed using Graph Pad Prism 6 (GraphPad Software, Inc., La Jolla, CA, USA).

## Results

**Figure 2:**
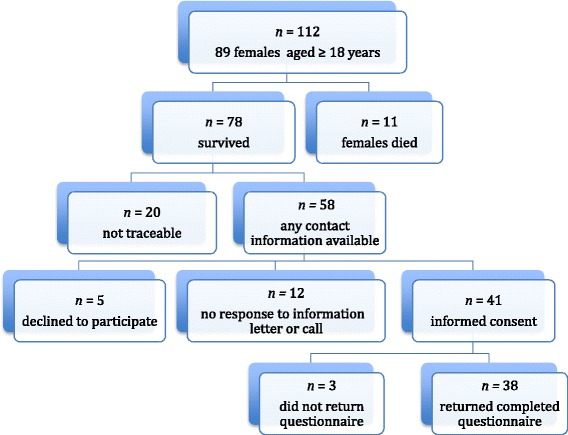
**Flowchart illustrating participant recruitment.**

Between 1970 and 1993, SCT resection has been performed in 112 children of whom 89 were female. Eleven of those 89 had died in the neonatal period or afterwards because of malignant transformation of the tumor; 20 patients could not be traced. Thus, 58 women were invited to participate. Five declined participation and twelve did not respond, maybe due to inability to deliver the information letter or because they were not willing to participate. Eventually, 41 women returned the signed consent form and received the questionnaire, of whom 38 returned the completed questionnaire (92.7%). A flow chart illustrating the participant recruitment is given in Figure 2.

### Patient characteristics

Data of 38 women were analyzed. Details about SCT surgery and tumor characteristics are listed in Table 1. Additional intra-abdominal operations were performed in five women: two underwent appendectomy, two underwent ureteral surgery during childhood with later an appendicovesicostomy in one and one woman was treated for urethral stricture. One woman had received adjuvant chemotherapy during childhood due to a malignant SCT. A total of thirteen women with a median age of 31.2 years (range 25.6 – 36.5 years) had become pregnant.

**Table 1:** **Altman classification** [6]**, tumor histology and operation details of women with or without offspring**

	Women treated for SCT* with offspring	Women treated for SCT* without offspring	***p -*** **value**
	***(n =*** **13** ***)***	***(n*** **= 25)**	
**Age**			
Median in years (range)	31.2 (25.6 – 36.5)	24.1 (18.5 – 41.2)	**< .001**
**Altman classification** [6]			
Altman type I or II	*n* = 8 (61.5%)	*n =* 18 (72.0%)	0.46
Altman type III or IV	*n* = 5 (38.5%)	*n =* 6 (24.0%)	
Unknown		*n =* 1 (4.0%)	
**Tumor histology**			
Benign	*n =* 10 (76.9%)	*n =* 20 (80.0%)	0.53
Malignant EST^#^	*n* = 1 (7.7%)	*n =* 4 (16.0%)	
Unknown	*n =* 2 (15.4%)	*n* = 1 (4.0%)	
**Coccyx resection**			
Yes	*n* = 11 (84.6%)	*n* = 22 (88.0%)	0.30
No	*n* = 1 (7.7%)	-	
Unknown	*n* = 1 (7.7%)	*n* = 3 (12.0%)	

*SCT = Sacrococcygeal teratoma, ^#^Malignant EST = malignant endodermal sinus tumor.

### Fertility and pregnancy

Twelve women became pregnant spontaneously; one became pregnant after in-vitro fertilization. The 25 women without offspring were significantly younger (median age 23.4 years, range: 18.4 – 41.1 years) than those with offspring and all reported that the childlessness was not involuntary.

Eight women were pregnant more than once with spontaneous abortion in two. The reason for the abortion was unknown. One woman had three abortions after a completed pregnancy. Her Altman type II SCT with mature histology had been completely removed with coccygeal resection with a sacral approach. The other woman with a spontaneous abortion was treated for Altman type I mature SCT that was also completely removed with the coccyx by a sacral approach. She had a successful pregnancy after a previous spontaneous abortion. No other complications during the pregnancies were reported.

### Delivery

**Figure 3:**
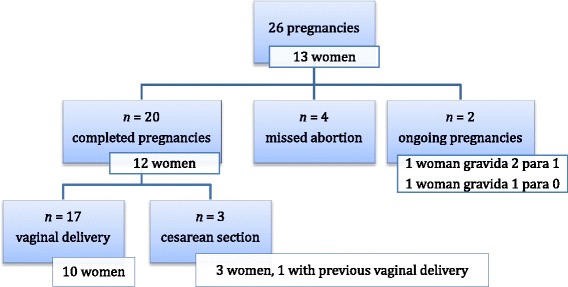
**Overview of the course of all pregnancies.**

Details of the course of all 26 pregnancies are shown in Figure 3. Of the 13 women who had become pregnant, a gynecologist performed pregnancy surveillance in six and a midwife in seven. Two of the women followed up by a gynecologist had been referred due to their previous history of SCT surgery. Twelve women with completed pregnancies gave birth to 20 infants of whom 17 were delivered vaginally, one by vacuum extraction. One of these women reported a complete anal sphincter rupture; the others only minimal perineal tears, or the perineum remained intact.

Conversion to a cesarean section was never necessary. Three women underwent a cesarean section, of whom one previously had a vaginal delivery. In two women this had been an emergency procedure due to deterioration of the child’s condition and not as a consequence of the prior SCT resection. In one the cesarean section had been planned because of previous intra-abdominal operations.

### Infants

The median gestational age of the infants was 39 weeks (range 30 weeks – 41 weeks); the median birth weight 3387 g (range 1156 g – 4620 g). Three infants were born prematurely. While the minority of the infants had a difficult (*n* = 2) or a moderate difficult (*n =* 1) start after the delivery, the majority had a good start (*n* = 17).

## Discussion

Sacrococcygeal teratoma is a neonatal tumor resected at young age [15]. The long-term outcome is relatively unknown and particularly pregnancy and delivery of women with prior SCT resection has been hardly addressed [16]. Our series of 20 deliveries is much larger than other series, however 20 deliveries remains a relatively small number.

Kohlberger and colleagues reported the case of a 23-year-old woman who had undergone resection during childhood of an Altman type II teratoma and had given birth to an infant by cesarean section. The cesarean section was needed due to insufficient descend of the infant during vaginal delivery. Post-delivery MRI imaging of the patient showed a dorsal bending of the sacrum with an area of scar tissue in front of it. The authors considered this anatomical transformation as an obstacle for vaginal delivery, even though the MRI scan showed a conjugate vera of 13.4 cm [12]. Others described conversion from vaginal delivery to a cesarean section in a 28-year-old woman, previously treated for SCT, as she had a rigid coccyx leading to a narrow pelvic outlet [13]. Nowadays, SCT surgery almost always included resection of the coccyx to achieve complete tumor resection [4]. We suppose that pelvic outlet obstruction during vaginal delivery is less relevant anymore. Recently, Shalaby and colleagues reported the obstetric outcome of two women previously treated for SCT who completed pregnancy. One gave birth to a healthy term baby by an uncomplicated vaginal delivery and in the other an emergency cesarean section was necessary due to the child’s condition [14].

The present study evaluates pregnancy and delivery in a cohort of 13 women who underwent SCT surgery during childhood. Our data suggests that these women can become pregnant spontaneously and that vaginal delivery is safe for mother and child, irrespective of SCT classification or tumor histology. In a relatively large proportion delivery was done by a midwife. However, it may be questionable of specialist delivery would not be preferable in a patient with previous pelvic surgery.

It has to be noted, that a presacral teratoma may be part of Currarino triad, which strictly seen is a different disorder but may have a similar clinical presentation. A Currarino triad consists of a presacral mass, sacral bone abnormalities and functional or anatomical rectal abnormalities. Moreover, myelum abnormalities including tethered cord or meningocele are frequently seen, which may affect delivery [15,17,18]. Therefore, we have not included these patients in our analysis.

None of the women who did not become pregnant reported involuntary childlessness. However the proportion of women without children appears to be relatively large compared to the proportion of women who gave birth to one or more children. The women without children were on average much younger than the women with children. Actually, in the Netherlands the age at which women become pregnant is relative high (mean 29.4 years) [19] and this may be the most logical explanation for this high proportion of women without children in our group as there are no differences in any of the other characteristics (Table 1).

One of the limitations of the study is the way the data were collected. Data were self- retrieved from patient records and self-reported by participants. This may have led to selection bias, as patients with problems or worse outcome may be less willing to report their problems and to complete the questionnaire. Still, the response rate was 92.7%, which suggests that any potential underestimation of problems, if present, is probably relative small. On the other hand, patients were not called back to the hospital and physical examination and imaging was not performed and thus we were not able to quantify the incidence of abnormal pelvic anatomy or functional changes. The questionnaire we used was ‘self-designed’. It included objective and measurable items giving exact information about pregnancy, delivery and birth. However, we deliberately did not ask for an exact Apgar score because we supposed that most participants would not be able to answer this question. We used a rather subjective scoring method (good – moderate – bad) to assess the neonatal condition after birth instead.

## Conclusions

Based on our results, we do not recommend routine cesarean section for women previously treated for SCT, as vaginal delivery seems to be safe in most cases for both mother and child. Cesarean section may be indicated for women with a medical history of multiple intra-abdominal operations as a consequence of the previous SCT surgery.

Health professionals involved in the care of these patients during pregnancy should be informed of the patient’s medical history in order to recognize problems at an early stage and to plan the optimal route of delivery individually.

## Abbreviations

SCT: Sacrococcygeal teratoma
Malignant EST: Malignant endodermal sinus tumor
